# Prevalence of Contraceptive Non-use Due to Husbands/Partners Influence Among Married Women in Ethiopia: A Multilevel Analysis Using Demographic and Health Survey 2016 Data

**DOI:** 10.3389/frph.2022.876497

**Published:** 2022-04-27

**Authors:** Melaku Hunie Asratie, Belayneh Ayanaw Kassie, Daniel Gashaneh Belay

**Affiliations:** ^1^Department of Women's and Family Health, School of Midwifery, College of Medicine and Health Sciences, University of Gondar, Gondar, Ethiopia; ^2^Department of Human Anatomy, College of Medicine and Health Sciences, University of Gondar, Gondar, Ethiopia; ^3^Department of Epidemiology and Biostatistics, Institute of Public Health, College of Medicine and Health Sciences, University of Gondar, Gondar, Ethiopia

**Keywords:** husband/partner, decision-making power, contraceptive, multilevel analysis, Ethiopia

## Abstract

**Background:**

In Ethiopia women with their husbands/partners are the decision-makers for contraceptives non-use suffered either due to the consequence of unintended pregnancy or due to the indirect impact of the secret use of contraceptives from their husbands/partners. Despite this challenge, there is a dearth of evidence about the magnitude of husbands/partners' decision-makers on contraceptives n non-used in Ethiopia.

**Objective:**

This study was aimed to assess the magnitude of husbands'/partners decisions on contraceptive non-use and associated factors among married and non-contraceptive user reproductive-age women in Ethiopia.

**Methods:**

The study was conducted based on Ethiopian demographic and health survey 2016 data which was a cross-sectional survey from 18 January 2016 to 27 June 2016. A total weighted sample size of 5,458 married and non-contraceptive user reproductive-age women were taken. A multilevel logistic regression model was used because of the data nature hierarchical, and variables with *p* ≤ 2 in the bivariable multilevel analysis were taken to multivariable multilevel analysis. Adjusted odds ratio with 95% CI was used to declare both the direction and strength of association and variables with *p* < 0.05 were considered statistically significant with the outcome variable.

**Results:**

Husband decision-making power on contraceptive non-use was 10.44% [9.65–11.28%]. Husband's educational level higher (adjusted odds ratio (AOR = 2.6; CI 1.4–4.7), being Muslim, protestant, and others in religion (AOR = 2.4; CI 1.7–3.5), (AOR = 2.1; CI 1.4–3.1), (AOR = 4.5; CI 2.3–8.5), respectively, media exposure (AOR = 1.4; CI 1.0–1.8), husband wants more children (AOR = 3.7; CI 2.8–4.8), husband desire did not know (AOR = 1.4; CI 1.1–1.9), information about family planning (AOR = 0.6; CI 0.4–0.8), visited by field worker (AOR = 0.7; CI 0.5–0.9), visited health facility (AOR = 0.6; CI 0.4–0.7), and community husband education high (AOR = 1.6; CI 1.1–2.4) were statistically significant with husband decision making power on contraceptive non-use.

**Conclusion:**

In Ethiopia 1 out of 10 married and non-pregnant women is influenced by their husband/partner's decision-making power of non-use contraceptives. Husband's educational level high, religion (Muslim, protestant, and others), media exposure, husband's desire for children (husband wants more and does not know), and community husband education were variables positively associated with the outcome variable; whereas having information about family planning, visited by field worker, and visited health facility were negatively associated husband decision making power for non-use contraceptive in Ethiopia.

## Background

Ethiopia is characterized by the highest fertility rate in Africa and with a large population in the age group under 15 years (1). Within this high fertility rate currently, Ethiopia focuses on the improvements of health system coverage for youthful population through different strategies like providing youth-friendly services and youth outreach related to sexual and reproductive health services (2, 3). There is also another effort that has been done in Ethiopia to stabilize this high fertility rate by adopting the innovative community health worker extension program which is highly active in the improvement of those maternal health indicators. Giving emphasis on accessibility of effective and affordable quality family planning is the priority agenda of the country among those indicators of improvement in maternal health care services (4, 5). In line with this priority agenda, all public health facilities of Ethiopia give family planning services free of charge (6), this affirms that the accessibility of family planning services for those with financial deficits is good. Despite those heroic efforts on family planning services in Ethiopia, there is high maternal mortality secondary to unintended pregnancy which can be tackled by scale-up family planning services (7).

Currently, the eventuation of unintended pregnancy is a challenge in Ethiopia. Women who had experienced unintended pregnancy mostly end up with unsafe abortions. In turn complication of unsafe abortion is one of the primary causes of maternal death in Ethiopia. There is evidence that shows 32% of all maternal death accounts are due to complications of unsafe abortion (8). The other bad terrain of unsafe abortion is the psycho-social impact due to stigma even though the women refrain from loss of life. Women who had committed unsafe abortions were mostly less acceptable by the community and always lived with the feeling of being criminal (9–11). All those mournful consequences of unintended pregnancy are directly associated with the unmet need demand for family planning services among reproductive age groups (12, 13). Only knowing the association of unmet need demand of reproductive age groups for family planning services with unintended pregnancy cannot alleviate the huge burden of maternal mortality either due to the impact of high fertility rate or due to direct complications of unintended pregnancy. Clear out the root causes of unmet need demand of reproductive age groups for family planning services and directly tackling those factors is the basic modality for the reduction of maternal mortality secondary to the problems (14). Unmet need for family planning service is an inclusive term that is defined as fecund women who want to postpone giving childbirth for more than 2 years or do not wants to give birth at all but she is not using a contraceptive. On the other hand, pregnant women either miss-timed or unwantedly or postpartum period women who had spent more than 12 months but did not use any contraceptive, in general, are considered an unmet need for family planning services (15). Within this definition, the prevalence of unmet needs among married reproductive-age women is 22% in Ethiopia in general and 24.08% specifically in the rural part (15, 16). Therefore, this high magnitude of contraceptives non-used among married reproductive-age women, despite the significant effort that has been given by the government to solve it still a problem in Ethiopia. Various kinds of literature had shown those possible determinant factors for this high prevalence of contraceptive not use among married reproductive age women like educational attainment, working status, age of marriage, wealth index, distance to reach a health facility, community poverty, community women education, community media exposure, residence, religion, knowledge on contraceptive, women autonomy, women decision making power, and attitude toward health care providers (16–22). From those findings, there is a research question that needs to be covered in timely, “whether husbands/partners' decision-making power for contraceptive non-use is high or low in Ethiopia and what are the factors associated with it?”

Intra-familial decision-making is a determinant factor for access to maternal health care services like family planning by women (23, 24). From the evidence, the decision-maker at the household level for contraceptive use can be women independently, husbands/partners independently, both women and husbands or, other relatives (25–27). When the decision-maker for contraceptive use is woman at the household level the probability to use contraceptive is high (28). There is also other evidence that shows contraceptive use coverage can be achieved through cultivating the culture of joint decision-making at the household level (27). The basic challenge is the involvement of husbands/partners in contraceptive use decision-making. Pieces of evidence done in sub-Saharan Africa showed that mostly husbands/partners were reluctant to contraceptive use decisions making (29). From clinical experience, women with husbands/partners are the primary decision maker for non-using contraceptives either may become pregnant unintended or need to take the contraceptive in a hidden manner. Taking contraceptives without the permission of their husbands/partners has multiple adverse consequences. The first challenge as women had explained in our clinical setup was difficult to select the most preferable contraceptive method from the perspective of side effects. To keep the secret those women mostly take injectable contraceptives as the first choice, and they are reluctant to use the most preferred contraceptive methods like implant and Jadel. The main reason that they had raised was injectable contraceptive is not visible for their husbands/partners once after it has been injected, whereas implants and Jadel are palpable to our arm by the husbands/partners. Even at the national level, the magnitude of injectable contraceptives among married reproductive-age women is high which is 23% whereas implants 8% as evidenced by EDHS 2016 report (15). But there is no published evidence about the reason for this high magnitude of the injectable contraceptive method with more side effects as compared to implants continuously reported for the health care providers by women.

As per the authors clinical experience, women who use contraceptives without the permission of their husbands/partners is they do not need to have healthcare provider consultancy and on-time treatment if contraceptive side effects in case happen. Because they fear disclosing to their husband unless conditions are suitable for her to access health care services secretly as usual. Despite all those challenges of husband decision-maker on contraceptive non-use, there is no study done yet, about the magnitude and its associated factors in Ethiopia, different studies had done about the magnitude of women's decision-making power on contraceptive use (30–32).

Therefore, this study aimed to assess the magnitude of husbands'/partners' decision-making power on contraceptives non-used and associated factors in Ethiopia. Doing this research can be an impute about the burden of not using contraceptives among married reproductive age women decided by husbands/partners and family planning-related program managers take as a baseline data to tackle culture-based prohibiting factors for contraceptive use. Furthermore, this finding can be a baseline data to do further research on the reasons for the decision of husbands/partners to non-use contraceptives qualitatively.

## Methods

### Study Design, Area, and Period

A cross-sectional study survey was done among reproductive-age women in Ethiopia from the 18 January 2016 to the 27 June 2016 by the Ethiopian central statistical agency (ECSA). In the case of our study, we have done a deep secondary analysis of the survey using EDHS 2016. Ethiopian demographic survey 2016 was the fourth survey conducted among nine regional states of the country (Tigray, Afar, Amhara, Oromia, Somali, Benishangul-Gumuz, Southern Nations Nationalities and People Region (SNNPR), Gambella, and Harari regions) and two city administrations (Addis Ababa and Dire Dawa). Ethiopia is an eastern African country that is the second-most populous country next to Nigeria. The federal level is divided into nine regions and two city administrations for administrative purposes, those regions are subdivided into zones, zones divided into woredas, and woredas divided into kebeles (the lowest administrative unit). Kebeles is also divided into census enumeration areas (EAs).

The details of the study area and study design are clearly elaborated from the document of the central statistical agency of Ethiopia (CSA) (33).

### Source Population

All reproductive age (15–49) women in Ethiopia were our source population.

### Study Population

**Figure 1:**
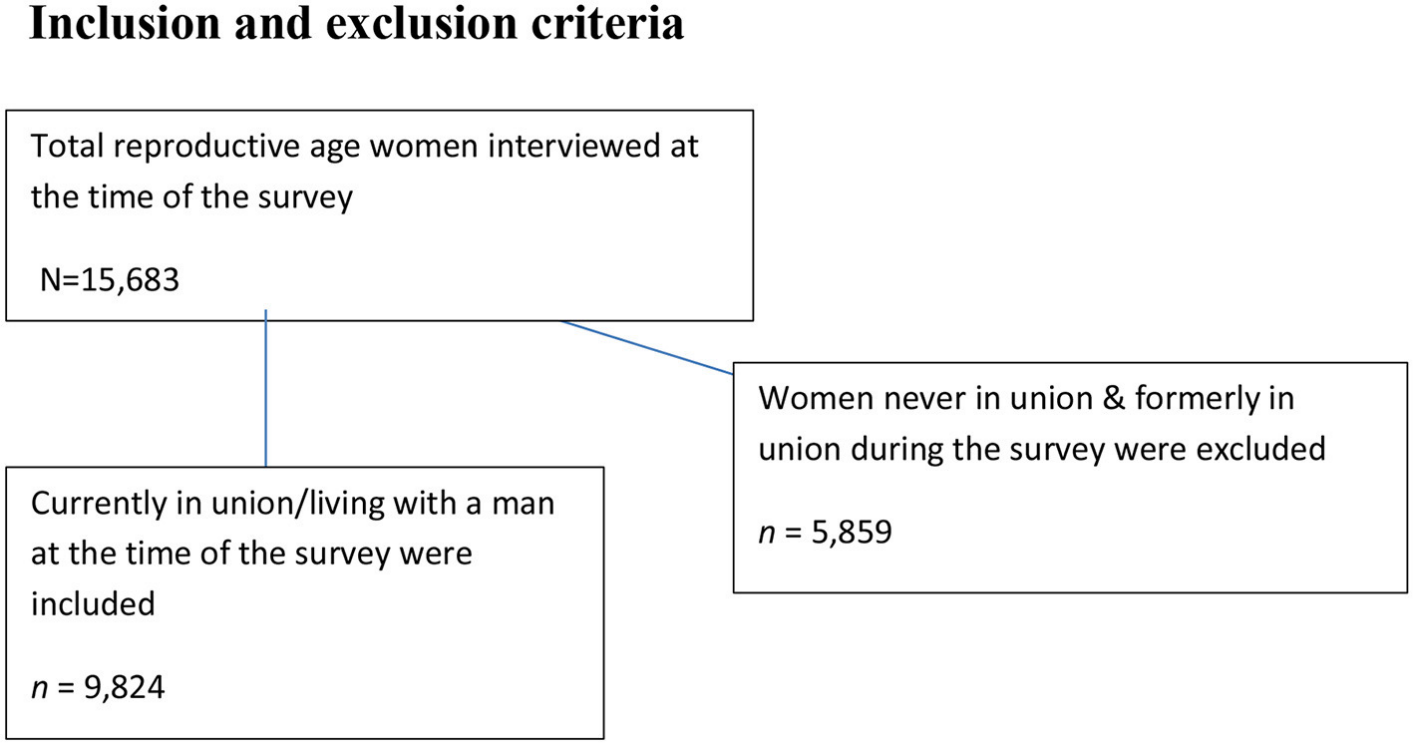
Inclusion and exclusion criteria.

All reproductive-age women who were married and not pregnant and not currently using any contraceptive methods were in the study population (Figure 1).

#### Sample Size Determination and Sampling Procedure

In EDHS, the survey was conducted with two stages stratified cluster sampling technique to select participants. In the 1st stage, 645 enumeration areas (EAs) were selected by stratifying into 202 urban areas and 443 rural areas. The sampling frame was the 2007 population and housing census using probability proportional to the EA (enumeration areas) scale. The details of the sampling procedure were elaborated in EDHS 2016 report from the Measure DHS website (www.dhsprogram.com). Finally, the weighted values were used for the analysis to keep the representativeness of the sampled data. Women's record (IR) EDHS data sets were used, and the sample size of this study was determined by using the variables V502, V213, and V312. By using the STATA command keeping V502 = 1 gives 9,824 married reproductive-age women, keeping V213 = 0 gives 8,734 married and non-pregnant women, and keeping V312 = 0 gives 5,754 married, non-pregnant, and non-contraceptive users women (34). Finally, we have used a weighting variable to attain 5,458 women who were married, not pregnant, and not currently using any kind of contraceptives.

### Study Variables

#### Dependent Variable

The outcome variable of this study was contraceptive non-use due to husbands/partners' influence. It was measured based on the woman's self-report of the decision-maker for contraceptive non-use at the time of the survey (husband/partner). The numerator was who makes the decision for non-use family planning (v632a) has four responses like respondent, husband/ partner, Joint decision, and other (34). Therefore, we have dichotomized it to Yes/No for the analysis.

#### Independent Variables

All those independent variables were grouped into three major classifications (socio-demographic factors, obstetrical-related factors, and health care service-related factors). During analysis of age of women in a year, age of husband/partner, women's and husband level of education, religion, current working status, wealth status, relation to household head, exposure to mass media, parity, number of under five age children, history of pregnancy termination, husband/partner desire for children, information about family planning services, knowledge on any kind of family planning, visited by a health worker in the past 12 months, field worker talked about family planning, visited a health facility in the last 12 month, at health facility talked about family planning and distance to health facility were variables moved as individual-level factors whereas residency, community-level women's education, community-level husband education, community-level poverty, and community-level media exposure were variables moved on community level factors (Table 1).

**Table 1:** Description and measurement of independent variables.

Age of women's	Re-coded into three categories with a value of “1” for 15–19, “2” for 20–34, and “3” for 35–49. In the data set this variable was continuous data.
Women's level of education	The variable women's educational level was recorded as no education primary, secondary, and higher in the dataset and we used without change.
Religion	Re-coded in four categories with a value of “1” for Orthodox, “2” for Muslim, “3” for protestant, and “4” for other religious groups (combining catholic, traditional and the other religious categories as most women's in this category are small in number).
Parity	In the dataset this variable was continuous data. We re-coded in to four categories with a value of “0” for nulliparous, “1” for Primiparous, “2” for multiparous and “3” for grand-multiparous.
Information about family planning	This variable was generated from four variables from the data set (1) heard about family planning from radio, (2) heard about family planning from newspaper/magazine, (3) heard about family planning from TV, (4) heard about family planning from text message. A women at least one from the four listed considered as informed.
Current working status	The variable current working status was recorded as Yes and No in the dataset and used was used without change for this study.
Wealth status	It was coded as “poorest,” “poorer,” “Middle,” “Richer,” and “Richest” in the EDHS data set. For this study we recoded it in to three categories as “poor” (includes the poorest and the poorer categories), “middle,” and “rich” (includes the richer and the richest categories).
Residence	The variable place of residence was recorded as “rural” and “urban” in the dataset and used was used without change for this study.
Community media exposure	Defined as the proportion of women who had mass media exposure within the cluster. The aggregate of individual women with mass media exposure can show overall mass media exposure of the cluster. It was categorized as high if cluster has more than or equal to median proportion (57.14%) of women with mass media exposure or low otherwise.
Community poverty	Defined as the proportion of women who resided in poor or poorest households within the cluster. The aggregate of individual households with poorest or poor wealth index can show overall poverty of the cluster. It was categorized as high if clusters had more than or equal to median proportion (60%) of poorest or poor households or low otherwise.
Community women's education	Defined as the proportion of women who attended primary/secondary/higher education within the cluster. The aggregate of individual woman's primary/secondary/higher educational level can show overall educational attainment of the women in the cluster. It was categorized as high if clusters with more than or equal to median proportion (27.27%) of primary/secondary/higher education or low otherwise.
Community husband partner education	Defined as the proportion of husbands/partners who attended primary/secondary/higher education within the cluster. The aggregate of individual husbands/partners primary/secondary/higher educational level can show overall educational attainment of the husband/partner in the cluster. It was categorized as high if clusters with more than or equal to median proportion (44.44%) of primary/secondary/higher education or low otherwise.

### Data Processing and Statistical Analysis

The data were 1st accessed from the website of http://www.measuredhs.com/ by online request of permission through a detailed explanation of our research purpose, and then the data were extracted, coded and both descriptive and analytical analyses were done by using statistical software STATA version 14. Statistical summaries like proportion and median were used to present descriptive statistics.

EDHS data had been collected by considering clusters as a study unit and this violet the independent assumptions of a standard logistic regression model. Therefore, multilevel logistic regression analysis was implemented. First, the intraclass correlation coefficient (ICC) of the null model was done to detect the presence of variation in the distribution of the outcome variable (decision making power of husband on contraceptive not use) among different clusters and the magnitude was 26.7% which entail us there is significant clustering effect that should be considered during analysis using an advanced statistical model. The median odds ratio (MOR) was also another indicator of the presence of a significant clustering effect with the value of 2.8(2.4–3.4) in the null model.

Fixed effects (a measure of association) were used to assess the relationship between the outcome variable and the independent variables. Crude odds ratio (COR) with a 95% confidence interval was used to measure both the direction and strength of the association. A variable with a *p* ≤ 2 was selected for the analysis in the adjusted model. Finally, in the multilevel analysis, the association between the outcome variable and explanatory variables was judged by using an adjusted odds ratio (AOR) with respect to a 95%CI, and statistical significance was declared at a *p*-value of <0.05.

Random effects (a measure of variability) were measured by intra-class correlation coefficient (ICC), median odds ratio (MOR), proportion change in variance (PCV), and deviance (-2 log-likelihood ratio).

Intra-class correlation coefficient (ICC): The value used to detect the variation in the distribution of outcome variable (decision making power of husband on contraceptive non-use) between clusters. In the null model, the ICC was 26.7% which means irrespective of other factors like socio-demographic, obstetrical, and health care service-related factors of our study cluster determine 26.7% of the variation in the distribution of the outcome variable.

Median odds ratio (MOR): Was used to quantify the middle odds ratio between the highest and the lowest odds ratio of the clustering effect. It is another way of quantifying cluster level variance into odds ratio. The MOR in the null model of this study was 2.8(2.4–3.4) which was significant. It was calculated as follows. MOR = exp. [√ (2 × VA) X0.6745], MOR = e0.95√VA where VA = cluster level variance.

Proportion change in variance (PCV): was used to explain the percentage of the variation in husband decision-making power on contraceptive non-use detected by the model with the available variables. The PCV of the final model of this study was 28% which means 28% of the variability was explained by the model that we fit whereas the rest 72% of the variability was not explained by the model.

Deviance −2 log-likelihood: Was used to measure the total variations that produce both the individual and community level factors. It was used for model comparison and the model with the lowest deviance was taken for the interpretation of the finding which was model IV.

## Results

### Socio-Demographic Characteristics of Women's and Husbands/ Partners

A total of 5,458 married, not pregnant, and who were not on contraceptives during the time of the survey were included in this study. Of those participants, 2,916 (53.3%) of them were within the age group of 20–34, 3,656 (67%) of husbands/partners aged was between 31 and 59 and 3,718 (68%) and 2,850 (52%) women and husband/partner had no formal education, respectively. Of all participants, 2,297(42%) of them were Muslim by religion, 3,942 (72%) of them had no work at the time of the survey, 2,494 (46%) of them were poor in wealth status, 4,815 (88%) of them were rural in residency and 4,376 (80%) of the participants were the wife of the household head. Women who had exposure to mass media were 3,625 (66%).

Of all participants, 2,732 (50.1%) women were low community education, 2,994 (55%) of the husbands of the participants were high community education, 3,513 (64%) of the participants were in low community poverty and 2,858 (52%) of the participants were at high community media exposure (Table 2).

**Table 2:** Socio demographic characteristics of women's and husbands/partners.

**Characteristics**	**Weighted frequency** **(*n* = 5458)**	**Percent**
**Age of women in year**		
15–19	313	5.7
20–34	2,916	53.3
35–49	2,229	41
**Age of the husband /partner**		
<31	1,327	24
31–59	3,656	67
>59	475	9
**Women's level of education**		
Had no formal education	3,718	68
Primary (grade 1–8)	1,370	25
Secondary (grade 9–12)	220	4
Higher	150	3
**Husband/partner level of education**		
Had no formal education	2,850	52
Primary (grade 1–8)	1,915	35
Secondary (grade 9–12)	414	8
Higher	279	5
**Religion**		
Orthodox	1,916	35
Muslim	2,297	42
Protestant	1,082	20
Others^*^	163	3
**Current working status**		
No	3,942	72
Yes	1,516	28
**Wealth status**		
Poor	2,494	46
Middle	1,088	20
Rich	1,876	34
**Residency**		
Urban	643	12
Rural	4,815	88
**Relation to house hold head**		
Head	650	12
Wife	4,376	80
Daughter	174	3
Others^**^	258	5
**Exposure to mass media**		
Yes	3,625	66
No	1,834	34
**Community women's education**		
Low	2,732	50.1
High	2,726	49.9
**Community husband education**		
Low	2,464	45
High	2,994	55
**Community poverty**		
Low	3,513	64
High	1,945	36
**Community media exposure**		
Low	2,600	48
High	2,858	52

Others^*^: *= Catholic, traditional follower,*

Others^**^: *= sister, mother in law*.

### Obstetrical Related Characteristics of Study Participants

Among 5,458 participants, 2,424 (44%) of them were multiparous, 1,712 (31%) of them with the number of children under 5 years age of 1, 4,783 (88%) of them had no history of pregnancy termination, and 1,551(38%) of participants husband/partner wants more children (Table 3).

**Table 3:** Obstetrical related characteristics of participants in Ethiopia.

**Variables**	**Frequency**	**Percent**
**Parity**		
Null Para	403	7
Primiparous	642	13
Multiparous	2,424	44
Grand multiparous	1,989	36
**Number of under five age children**		
No children	1,513	28
1	1,712	31
2	1,657	30
≥3	576	11
**History of pregnancy termination**		
No	4,783	88
Yes	675	12
**Husband desire for children**		
Both wants same	1,903	35
Husband wants more	1,551	38
Husband wants fewer	386	7
Don't know	1,618	30

### Health Care Services Related Characteristics of Participants

Among all participants, 4,189 (77%) of the participants had gotten information about family planning, 5,353 (98.1%) of them were knowledgeable about at least one type of family planning method, and 4,034 (74%) of them were visited by a health worker within the last 12 months of before the time of the survey. Of 1,424 respondents, 844 (59%) of them were talked with field workers about family planning and from 2,385 respondents 1,527 (64%) of them talk about family planning at the health facility. Among all respondents of this study 3,297 (60%) of them were at big problem ofreaching the nearby health facility (Table 4).

**Table 4:** Health care services related characteristics of participants in Ethiopia.

**Variables**	**Frequency**	**Percent**
**Information about family planning**		
No	4,189	77
Yes	1,269	23
**Knowledge on family planning methods**		
No	105	1.9
Yes	5,353	98.1
**Visited by health worker within 12 month**		
No	4,034	74
Yes	1,424	26
**Did field worker talk about family planning (*n* = 1,424)**		
No	590	41
Yes	844	59
**Did you visit health facility within 12 month**		
No	3,074	56
Yes	2,384	44
**At health facility have you talked about family planning (*n* = 2,385)**		
No	1,527	64
Yes	858	36
**Distance to reach health facility**		
Not a big problem	2,161	40
A big problem	3,297	60

### Prevalence of Husband/Partner Decision Making Power on Contraceptive Non-Use

**Figure 2:**
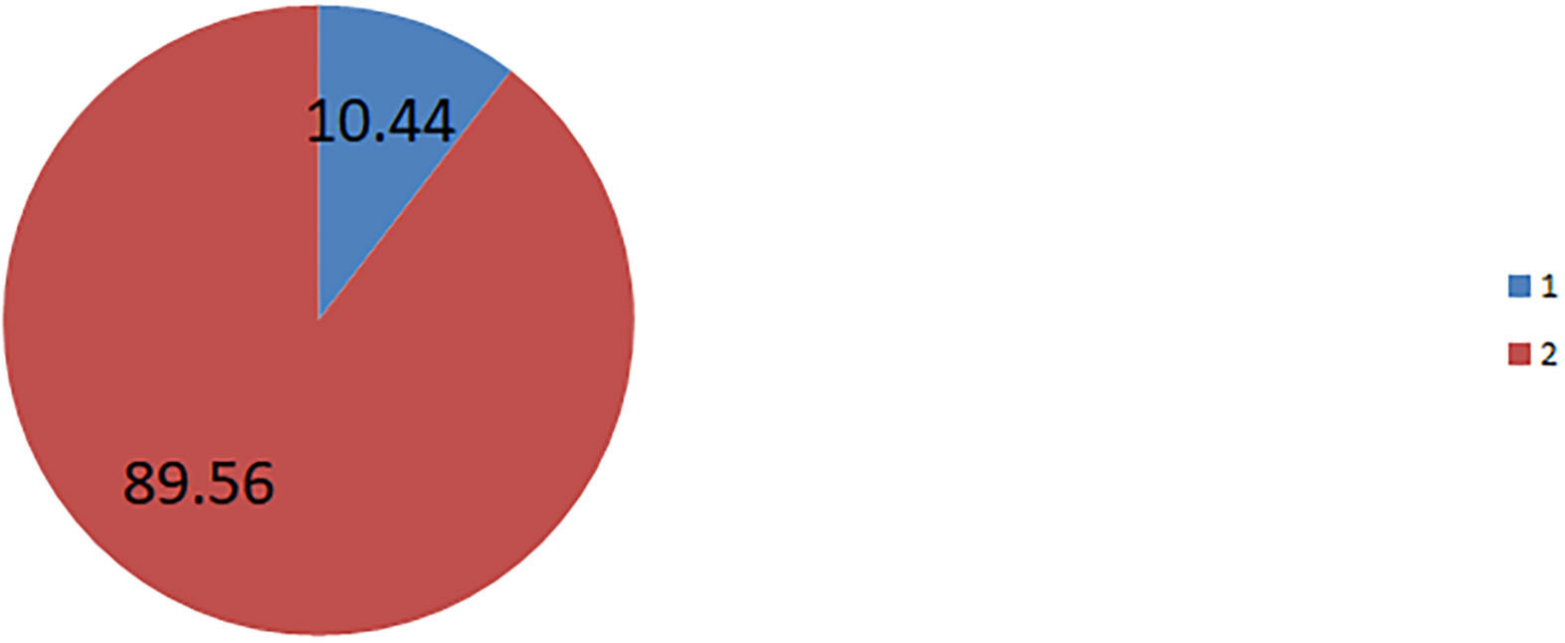
The prevalence of husband/partner decision making power on contraceptive non-use, in Ethiopia.

The prevalence of husband decision-making power on contraceptive non-use among married, non-pregnant, and non-contraceptive user reproductive-age women in Ethiopia was found to be 10.44%; 95%CI 9.65–11.28 (Figure 2).

Multilevel logistic regression analysis of determinant factors for decision making power of husbands/partners on contraceptive non-use among reproductive age, married, non-pregnant, and non-contraceptive user women in Ethiopia, secondary analysis of EDHS 2016.

There was a total of 18 variables candidates for the adjusted model with a *p*-value of ≤0.2 after assessing the association with the outcome variable independently. A total of seven individual-level factors (husband educational level, religion, media exposure, husband/partner desire for children, information about family planning, visited by field worker, and visited health facility were significantly associated with the outcome variable (decision making power of husbands on contraceptive non-use) in the model I and those variables were continued significantly in the final model which is model IV. In the 3rd model, only one community-level variable (community husband education) was significantly associated with the outcome variable and it was continued significantly associated in the final model which is model IV. In the final model (model IV) eight variables both individual and community levels factors were statistically significant with the outcome variable decision-making power of husband/partner on contraceptive non-use among non-pregnant and non-contraceptive use married reproductive-age women.

Participants with a husband/partner educational status of higher were 2.6 times more likely to decide their husband/ partner on non-using contraceptives (AOR = 2.6; 95% CI 1.4–4.5) as compared to those participants with husbands/partners who had no formal education. Women who were Muslim, protestant, and other by religion were 2.4, 2.1, and 4.5 more likely to decide their husband/partner on non-using contraceptives (AOR = 2.4; 95% CI 1.7–3.3), (AOR = 2.1; 955 CI 1.4-3.4), (AOR = 4.5; 95% CI 2.3–8.5) as compared to women who were orthodox by religion, respectively.

Women who had mass media exposure were 1.4 times more likely to decide their husband/partner on non-using contraceptives (AOR = 1.4; 95% CI 1.0–1.8) as compared to women who had no mass media exposure. Participants with their husbands/partners were desired children were 3.7 times more likely to decide their husband on non-using contraceptives (AOR = 3.7; 95% CI 2.8–4.8) as compared to participants with husbands/ partners and women want the same. Women with the response of I do not know about the desire of their husband/partner on children are 1.4 times more likely to decide their husband/ partner on contraceptive non-use (AOR = 1.4; 95% CI 1.1–1.9) as compared to both women and husband/partner want the same.Women who had been visited by field workers were 30% less likely to decide their husband/partner on contraceptive non-use (AOR = 0.7; 95% CI 0.5–0.9) as compared to women who had not been visited by the field workers. Women who had visited health facilities within the last 12 months before the survey were 40% less likely to decide their husband/partner on contraceptive non-use (AOR = 0.6; 95% CI 0.4–0.7) as compared to those who had not visited on the period. Women with their husband/partner community education were high 1.6 times more likely to decide their husband/ partner on contraceptive non-use (AOR = 1.6; 95% CI 1.0–2.4) as compared to those women with their husband/partner at a low community education level (Table 5).

**Table 5:** Multilevel logistic regression analysis of determinants of husband decision making power on contraceptive non-use in Ethiopia, EDHS 2016.

**Variable**	**Null Model**	**Model II**	**Model III**	**Model IV**
		**AOR(95% CI)**	**AOR(95% CI)**	**AOR(95% CI)**
**Age of women**				
15–19		1		1
20–34		1.3 (0.7–2.2)		1.3 (0.7–2.2)
35–49		0.8 (0.5–1.5)		0.8 (0.5–1.5)
**Women educational level**				
No formal education		1		1
Primary (grade 1–8)		0.86 (0.6–1.1)		0.8 (0.7–1.2)^***^
Secondary (grade 9–12)		0.9 (0.4–1.8)		0.9 (0.5–1.9)^***^
Higher		0.4 (0.15–1.1)		0.5 (0.2–1.3)^***^
**Husband educational level**				
**No formal education**		1		1
Primary (grade 1–8)		0.8 (0.6–1.1)		0.8 (0.6–1.0)
Secondary (grade 9–12)		1.4 (0.8–2.2)		1.3 (0.8–2.1)
Higher		2.5 (1.4–4.6)		2.6 (1.4–4.7)^***^
**Religion**				
Orthodox		1		1
Muslim		2.4 (1.7–3.5)		2.4 (1.7–3.5)^***^
Protestant		2.3 (1.6–3.4)		2.1 (1.4–3.1)^**^
Others^*^		4.8 (2.5–9.2)		4.5 (2.3–8.5)^***^
**Relation to house hold head**				
Head		1		1
Wife		1.2 (0.9–1.9)		1.3 (0.9–1.7)
Daughter		0.5 (0.25–1.3)		0.6 (0.3–1.3)
Others^**^		0.5 (0.2–1.0)		0.5 (0.3–1)
**Wealth status**				
Poor		1		1
Middle		0.9 (0.7–1.33)		0.9 (0.7–1.3)
Rich		0.8 (0.6–1.1)		0.8 (0.6–1.1)
**Media exposure**				
No		1		1
Yes		1.4 (1.1–1.8)		1.4 (1.0–1.8)^***^
**Husband desire for children**				
Both wants same		1		**1**
Husband wants more		3.7 (2.8–4.9)		3.7 (2.8–4.8)^***^
Husband wants fewer		1.1 (0.6–1.8)		1.1 (0.7–1.8)
Don't know		1.4 (1.1–1.9)		1.4 (1.1–1.9)^***^
**Information about family planning**				
No		1		1
Yes		0.6 (0.44–0.8)		0.6 (0.4-0.8)^**^
**Visited by field worker**				
No		1		1
Yes		0.7 (0.5–0.9)		0.7 (0.5-0.9)^***^
**Distance to reach health facility**				
Big problem		1		1
Not big problem		0.8 (0.6–1.1)		0.9 (0.7–1.1)
**Knowledge on family planning methods**				
No		1		1
Yes		0.6 (0.4–1.2)		0.6 (0.3–1.1)
**Visited health facility**				
No		1		1
Yes		0.6 (0.5–0.8)		0.6 (0.4-0.7)^***^
**Residency**				
Urban			1	1
Rural			1.8 (1.1–3.3)	1.7 (0.9–3.1)
**Community women education**				
Low			1	1
High			0.6 (0.4–1.0)	0.8 (0.5–1.1)
**Community husband education**				
Low			1	1
High			1.7 (1.2–2.7)	1.6 (1.0-2.4)^***^
**Community poverty**				
Low			1	
High			1.5 (1.0–2.2)	1.0 (0.7–1.6)
**Community media exposure**				
Low			1	1
High			0.9 (0.6–1.4)	1.1 (0.7–1.6)
**Random effect**				
Community level variance	1.2	0.91	1.09	0.86
ICC	26.7%	21.8%	24.8%	20.8%
MOR	2.8 (2.4–3.4)	2.47	2.69	2.41
PCV	Reference	24%	9%	28%
**Model fit statistics**				
Log likely hood	−1711.7596	−1578.7052	−1700.8349	−1573.1868
Deviance	3423.5192	3157.4104	3400	3146.3736

*AOR, Adjusted Odds Ratio; CI, Confidence Interval; MOR, Median Odds Ratio;*

Other*: *Catholic, traditional follower;*

others**: Mother-in-low, sister, other relatives; PCV, Proportion Change in Variance; *, p-value < 0.05;

** *= p-value < 0.01;*

*** *= p-value < 0.001 for bold value represent just to give emphasis*.

## Discussion

Decision-making power in accessing family planning services is the determinant for hindering the tragic effect of unintended pregnancy on the health of the women, the neonate, and the child as well (29, 35). Improving the decision-making power of reproductive-age women on their contraceptive usage can reduce the direct causes of maternal loss, like unsafe abortion or unintended pregnancy (36). Unintended pregnancy is mostly associated with a contraceptive unmet need, which is the direct output of the lack of decision-making power of women on contraceptive use in Ethiopia (17). Decision-making power on contraceptive use of married reproductive age women in Ethiopia is overtaken either by themselves independently, jointly with their husband/partner, with other relatives, or only by their husbands/partners independently (37). Husband/ partner decision-maker on contraceptive use of married reproductive-age women is unacceptable decision-making power as the husbands/partners mostly influence them to non-use contraceptives.

Despite this scientific evidence on the impact of husband decision-making power on contraceptive utilization, there is no research yet done about the magnitude of husband/partner decision-maker on contraceptive non-use. How much married, non-pregnant, and non-contraceptive users' reproductive-age women were influenced by their husband/partner for non-use was not given an answer yet. Therefore, this study was done to assess the prevalence of husband/partner decision-makers on contraceptives non-used in Ethiopia from a secondary analysis of EDHS 2016.

The prevalence of husband/partner decision-maker on contraceptive non-use among reproductive-age married, non-pregnant, and currently not on contraceptive use in this study is 10.44% [9.65–11.28%]. This is an informative finding which means 1/10 of reproductive age, married, non-pregnant, and currently not using contraceptives woman did not use contraceptives due to the influence of their husband/partner. The possible explanation could be that the study population in this study was fixed by considering those women who were not pregnant as criteria. Therefore, as they were not pregnant husbands/partners might influence them to non-use contraceptives due to the desire of having children. In turn, the husband/partner's desire for children was one individual-level factor that was significantly associated with the outcome variable (husband/partner decision-making power on contraceptive non-use) in this study.

Related to the factors associated with husband/partner's decision making power on contraceptive non-use, seven individual-level factors (husband's educational level, religion, mass media exposure, husband's desire for children, information about family planning, visited by field worker, visited health facility) and one community-level factor (community husband education) were significantly associated with the outcome variable in the final model which is model four.

Women whose husbands/ partners were at a higher educational level were 2.6 times more likely to decide their husbands/partners on contraceptive non-use as compared to those women with their husbands/partners who had no formal education. An explanation could be those husbands/partners with higher educational levels are more knowledgeable about contraceptive side effects, especially on the adverse effects on maternal health. Therefore, they prefer to use natural family planning methods by educating their wives. There is evidence that shows the educated population needs to cultivate the culture of natural family planning method with its limitation due to the introduction of side effects of artificial contraceptives (38). Women who were Muslim in religion were 2.4 times more likely to decide their husbands/partners on contraceptives non-use as compared to those women orthodox by religion. This finding was supported by evidence as religion prohibits women from using contraceptives in general irrespective of which type of religion (39). n the other hand, there is a finding that directly shows that being Muslim in religion is one factor for contraceptives not being used as compared to other types of religions (40). The possible explanation could be those Muslims are smaller in size as compared to orthodox followers and they need to compute by increasing fertility rate from the experience of real-world and contraceptive use opposition among Muslim society is extraordinarily strong to orthodox (41, 42).

Women who are protestant are 2.1 times more likely to decide their husbands/partners on contraceptives non-used as compared to those women orthodox by religion. The possible explanation could be that women with Protestantism in religion are highly interested to give birth to preachers that substitute them. There is evidence of those religious types with small numbers their first strategy is to increase the number of preachers (43). Women who had media exposure were 1.4 times more likely to decide their husbands/partners on contraceptives non-use as compared to those women who had no media exposure. An explanation could be those women who had exposure to media might be knowledgeable about contraceptive use therefore the reason for not using contraceptives could be their husband/partner's decision despite their great desire to use. But those who had no exposure might not have enough knowledge about contraceptives and they might decide either by themselves or jointly with their husband for contraceptive non-use. There is evidence that shows women with mass media exposure are highly interested in contraceptive use unless their husband prohibits them (28).

Women with husbands/partners who desired more for children were 3.7 times more likely to decide their husbands/partners on contraceptive non-use as compared to those who both want the same. The possible explanation could be due to excessive desire can be achieved by a high fertility rate in turn this might lead to less utilization of contraceptives. Women who did not know the desire their husbands/partners for more children were 1.4 times more likely to decide their husbands/partners on contraceptive non-use as compared to those who both want the same. An explanation could be that most decision-maker husbands/partners about any type of health services their desire is hidden from their wives. Because they are not open to their wives, therefore, they might independently decide even on contraceptives non-used.

Women who were informed about family planning services were 40% less likely to decide their husbands/partners on contraceptives non-used as compared to those women who were not informed. The possible explanation could be that most informed women are highly cooperative with their husbands/partners and they have the capability to convince them. Therefore, mostly the status of contraceptive usage cannot be shrouded by the independent decision-making power of their husbands/partners they can develop joint decision-making power. Women who had been visited by field workers were 30% less likely to decide their husbands/partners on contraceptives non-use as compared to those women who did not visit by the field workers. The possible explanation could be that when field workers visit the community; they might provide substantial community education about women empowerment and the concept of decision-making power at the community and household level. This in turn builds the culture of contraceptive use though the ordinary agreement between the husbands/partners and women. This justification was supported by evidence as community engagement of health care providers as a form of fieldwork increases women's decision-making power at the community and household level can be improved (44, 45). Women who had visited health facilities within the last 12 months before the survey were 30% less likely to decide their husbands/partners on contraceptive non-use as compared to those women who did not visit within the specified period. The possible explanation could be mostly those women who had visited health facilities are the primary decision-maker at the household level to access any health care service. There is evidence that shows among all participants who come to the health facility majority of them were the decision made by themselves (23). Therefore, those women who can decide to reach health facilities might also decide by themselves on contraceptive use or non-use.

Women with community husbands'/partners' education were high 1.6 times more likely to decide their husbands/partners on contraceptive non-use as compared to those women with husbands'/partners' community education were low. The possible explanation could be due to knowledge of the side effects of contraceptives among those educated groups might create negativity and prohibit their wives from contraceptives. This evidence was elaborated above for individual-level factor which is the husband's educational level directly associated with the prohibition of contraceptive use.

### Strengths and Limitations of This Study

This study used nationally representative data, which were collected with standardized and validated data collection tools.

This study used an advanced model that accounts for the correlated nature of the Ethiopian Demographic and Health Survey (EDHS) data in the determination of estimates.

The cross-sectional nature of the survey does not show the temporal or causal relationship between independent variables and the outcome variable.

## Conclusion

In Ethiopia 1 out of 10 married and non-pregnant women is influenced by their husband/partner's decision-making power of non-use contraceptives. Husband's educational level high, religion (Muslim, protestant, and others), media exposure, husband's desire for children (husband wants more and does not know), and community husband education were variables positively associated with the outcome variable; whereas having information about family planning, visited by field worker, and visited health facility were negatively associated husband decision making power for non-use contraceptive in Ethiopia.

## Data Availability Statement

The original contributions presented in the study are included in the article/supplementary material, further inquiries can be directed to the corresponding author.

## Ethics Statement

Since the study was a secondary data analysis of publicly available survey data from the MEASURE DHS program, Ethical Approval and participant consent were not necessary for this particular study. We requested DHS Program and permission was granted to download and use the data for this study from http://www.dhsprogram.com. The Institution Review Board approved procedures for DHS public-use datasets do not in any way allow respondents, households, or sample communities to be identified. There were no names of individuals or household addresses in the data file. The geographic identifiers only go down to the regional level (where regions are typically very large geographical areas encompassing several states/provinces). Each enumeration area (Primary Sampling Unit) has a PSU number in the data file, but the PSU numbers do not have any labels to indicate their names or locations.

## Author Contributions

MA initiated the idea of research, conceptualized, developed proposal, did the analysis, and wrote the manuscript. DB did the analysis. DB and BK reviewed the manuscript and corrected it. Finally, all the authors have approved the manuscript, by preparing it for submission.

## Conflict of Interest

The authors declare that the research was conducted in the absence of any commercial or financial relationships that could be construed as a potential conflict of interest.

## Publisher's Note

All claims expressed in this article are solely those of the authors and do not necessarily represent those of their affiliated organizations, or those of the publisher, the editors and the reviewers. Any product that may be evaluated in this article, or claim that may be made by its manufacturer, is not guaranteed or endorsed by the publisher.
